# The Influence of Plant Litter on Soil Water Repellency: Insight from ^13^C NMR Spectroscopy

**DOI:** 10.1371/journal.pone.0152565

**Published:** 2016-03-29

**Authors:** Gaspare Cesarano, Guido Incerti, Giuliano Bonanomi

**Affiliations:** Dipartimento di Agraria, University of Naples Federico II, via Università 100, 80055, Portici, Naples, Italy; Old Dominion Univ., UNITED STATES

## Abstract

Soil water repellency (SWR, i.e. reduced affinity for water owing to the presence of organic hydrophobic coatings on soil particles) has relevant hydrological implications because low rates of infiltration enhance water runoff, and untargeted diffusion of fertilizers and pesticides. Previous studies investigated the occurrence of SWR in ecosystems with different vegetation cover but did not clarify its relationships with litter biochemical quality. Here, we investigated the capability of different plant litter types to induce SWR by using fresh and decomposed leaf materials from 12 species, to amend a model sandy soil over a year-long microcosm experiment. Water repellency, measured by the Molarity of an Ethanol Droplet (MED) test, was tested for the effects of litter species and age, and compared with litter quality assessed by ^13^C-CPMAS NMR in solid state and elemental chemical parameters. All litter types were highly water repellent, with MED values of 18% or higher. In contrast, when litter was incorporated into the soil, only undecomposed materials induced SWR, but with a large variability of onset and peak dynamics among litter types. Surprisingly, SWR induced by litter addition was unrelated to the aliphatic fraction of litter. In contrast, lignin-poor but labile C-rich litter, as defined by *O*-alkyl C and N-alkyl and methoxyl C of ^13^C-CPMAS NMR spectral regions, respectively, induced a stronger SWR. This study suggests that biochemical quality of plant litter is a major controlling factor of SWR and, by defining litter quality with ^13^C-CPMAS NMR, our results provide a significant novel contribution towards a full understanding of the relationships between plant litter biochemistry and SWR.

## Introduction

Water repellency (WR) is a property of soil that reduces its affinity for water owing to the presence of hydrophobic coatings on soil particles [1]. In nature, soil hydrophobicity is highly variable, such that soils resist wetting for periods ranging from few seconds to weeks [2], with relevant hydrological and geomorphologic implications. Among these, reduced rates of infiltration due to high soil water repellency (SWR) enhance water runoff rates, which in turn can increase erosion risk [3] and untargeted diffusion of fertilizers and pesticides into surficial water flows and groundwater resources [2,4]. Moreover, SWR by modifying water availability indirectly affects seed germination, seedling establishment and plant growth both in natural and agro-ecosystems [5]. However, SWR can lead also to positive effects at ecosystem level, being associated with the stability of soil aggregates and thus with carbon sequestration in soil [1,6].

The origin and severity of SWR depends on several processes, affected by both chemical-physical and biological factors. Soil texture, aggregation state, pH, moisture and mineral composition of the clay fraction [7], as well as wildfire occurrence [8], are considered the main abiotic determinants. On the other hand, hydrophobic compounds inducing SWR can be produced by fungal and bacterial activity [9], exuded by plant roots [10] or released during litter decomposition [11,12]. The rates of such biological processes are highly species-specific. Therefore, the formation, intensity and persistence of SWR is highly affected by the specific composition of the overlying plant community [2,13,14]. Tree species have been often associated with SWR, as in the cases of eucalyptus [15], pines [16] and oaks [17]. However, soil hydrophobicity has been also found under shrubs in different ecosystems including heathlands [14], Mediterranean maquis [17,18], and agroecosystems [11]. In a multi-species comparative analysis investigating soil hydrophobicity in semi-arid conditions, Mataix-Solera & Arcenegui [19] observed higher SWR frequency under trees of *Pinus halepensis* and *Quercus coccifera* compared to the shrubs *Juniperus oxycedrus* and *Rosmarinus officinalis*. Such pattern was negatively associated with soil pH and positively with organic matter content in soil. The latter association was also reported in the rangelands of Extremadura [20] in soil samples collected under *Quercus ilex*, and, to a lesser extent, under *Retama sphaerocarpa*, *Quercus suber*, and different grasses. The positive association between SWR and organic C content has been attributed to different chemical compounds released in soil either by plant litterfall, or during the decomposition process, including resins, waxes, phenolic compounds and aromatic oils [2,14,21]. Taken together, all these observations suggest that, in field conditions, litter biochemistry is a main determinant of SWR occurrence and persistence. However, despite such evidences, the relationships between litter biochemical quality and SWR has been rarely investigated (but see [11]). In this perspective, several chemical throughput methods are currently available and have been applied to collect direct information on the characteristics of organic matter, including pyrolysis-gas chromatography/mass spectrometry [22], near infrared reflectance spectroscopy [23] and ^13^C-cross-polarization magic angle spinning (CPMAS) nuclear magnetic resonance (NMR) spectroscopy [24,25]. In detail, ^13^C-CPMAS NMR has been proven useful to provide a description of the total organic chemical composition of complex matrices, such as plant litter [24], and its relationships with decay rate [26] and plant growth inhibition [27].

In this study we used a detailed litter characterization by ^13^C-CPMAS NMR in solid state [24], coupled with bioassays in microcosms, to investigate the effects of litter biochemical quality on litter and soil water repellency. In detail, we evaluated the capability of 24 litter types, spanning a wide range of biochemical quality, to induce SWR. Specific aims of the study were to:

1. assess the water repellency of different litter types;
2. describe the long-term dynamics of SWR, after induction by litter application;
3. explore the relationships between SWR and litter biochemical quality, as defined by ^13^C-CPMAS NMR spectroscopy and standard chemical metrics.

## Materials and Methods

### Litter collection, decomposition experiment, and chemical analyses

Litter materials used in the present study derive from a previous litterbag decomposition experiment focused on litter mass loss dynamics [26]. Leaves of twelve plant species including two perennial grasses (*Ampelodesmos mauritanicus* and *Festuca drymeia*), two evergreen shrubs (*Arbutus unedo* and *Coronilla emerus*), one vine (*Hedera helix*), four evergreen trees (*Cupressus sempervirens*, *Picea excelsa*, *Pinus halepensis* and *Quercus ilex*) and three deciduous trees (*Castanea sativa*, *Populus nigra* and *Robinia pseudoacacia*) were selected to represent a wide range of litter quality. Freshly abscissed leaves were collected in the field from nets placed under the plants. Leaves were air dried at room temperature in laboratory until a constant weight was reached and then stored at room temperature.

The litter decomposition experiments were carried out in microcosms according to the litterbag method [28]. Large (20 x 20 cm^2^) terylene litterbags (mesh size 2 mm) were filled with 6 g of dry leaf litter and placed inside trays (100 x 100 x 30 cm^3^). Microcosms were kept in a growth chamber under controlled temperature (18±2°C night and 24±2°C day) and watered every seven days to field capacity with distilled water. Litterbags (10 replicates) were harvested after 180 days of decomposition for a total of 120 litterbags (12 species x 1 sampling dates x 10 replicates). Bags were dried in the laboratory at room temperature until reaching constant weight and the remaining material weighed. In this way, 24 organic materials (12 plant species at two litter ages) were produced: fresh undecomposed litter (thereafter indicated as 0 days) and litter decomposed for 180 days.

Biochemical quality of the 24 materials was previously reported [26]. Briefly, ^13^C-CPMAS NMR analyses of the litter samples showed a rapid reduction of carbohydrates (spectral regions corresponding to di-*O*-alkyl C and *O*-alkyl C) and a progressive increase of alkyl C and methoxyl C, as decomposition was proceeding [26,29]. These data were used as a reference dataset for biochemical quality of plant residues, and related to WR of pure litter as well as of soil amended with litter. The 24 materials were characterized for total C and N contents, labile C, proximate cellulose and lignin content [30] and by spectral data from ^13^C-CPMAS NMR in solid state (for details see [26]). Selection of spectral regions and identification of corresponding classes of C-types were performed according to Bonanomi et al. [26], and previous studies [24,31–34]. The following seven regions and C types were considered: 0–45 ppm = alkyl C; 46–60 ppm = N-alkyl and methoxyl C; 61–90 ppm = *O*-alkyl C; 91–110 ppm = di-*O*-alkyl C; 111–140 ppm = H- and C- substituted aromatic C; 141–160 ppm O-substituted aromatic C (phenolic and O-aryl C); 161–190 ppm carboxyl C.

### Litter water repellency assessment

The Molarity of an Ethanol Droplet (MED) test [20,35] was utilized to measure litter WR. This method reports the lowest volume percent concentration of an ethanol solution, in a range between 0% and >36%, that is absorbed by the soil sample within 5 s. Five drops (20 μl) of distilled water (0% ethanol) were placed over a layer of dry litter (thickness of 1 cm) placed in a 9 cm Petri dish. If the drops were absorbed within 5 s, we recorded a MED value of 0 (i.e. percentage of ethanol of 0%). Conversely, if distilled water drops were not absorbed within 5 s, the procedure was recursively repeated with a progressively increased ethanol concentration (1, 3, 5, 8.5, 13, 18, 24, 36%) annotating, as MED value, the lower concentration at which at least 5 drops were absorbed by the substrate in less than 5s. Finally, if drops at 36% ethanol were not absorbed, the highest MED index value (>36% ethanol) was recorded.

The water repellency of each tested litter material (LWR) was classified based on the resulting MED value, according to an ordinal scale deeming hydrophilic the samples with MED value of 0 (i.e. pure water), whereas the samples with MED values > 0 were judged hydrophobic at a progressively higher degree (S1 Table).

### Effect of plant litter on soil water repellency: a microcosms experiment

The effect of plant litter on SWR was assessed with laboratory microcosm experiments. Experimental microcosms were placed in a growth chamber under controlled conditions of temperature (18±2°C night and 24±2°C day). Petri dishes (diameter of 9 cm) were filled with 10 g of air dried sandy soil collected in a Mediterranean maquis where some of the litter samples had been collected. The soil was a sandy loam soil, (sand 66.1%, silt 16.9%, clay 17.0%) with pH = 7.65, organic C = 12.0 g kg^-1^, total N = 0.64 g kg^-1^, total CaCO_3_ = 224 g kg^-1^, available phosphorus (P_2_O_5_) = 28.3 mg kg^-1^, exchangeable K = 0.13 meq 100 g^-1^, exchangeable Mg 5.11 meq 100 g^-1^, exchangeable Ca 18.6 meq 100 g^-1^, exchangeable Na 0.40 meq 100 g^-1^, and electric conductivity (EC) = 0.17 dS m^-1^. Samples in Petri dishes were amended with dry powdered leaf litter at 2% (dry weight) and then mixed to obtain a homogeneous soil-litter distribution. Experimental values of litter addition were within the range observed in natural ecosystems, considering the amount of litterfall and standing litter [36]. A microbial inoculum was prepared by mixing 90 g of water with 10 g of the sandy soil collected from the topsoil layer (depth 0 to -10 cm from ground level). The inoculum was sprayed inside the microcosms in order to enhance the start up of the decomposition process. Petri dishes were harvested after 1, 3, 5, 10, 30, 50, 100 and 300 days of incubation for a total of 2,000 experimental units (24 litter types x 8 sampling dates x 10 replicates, plus the unamended control), air dried at room temperature and then stored. Soil water repellency was assessed as described in the previous section.

### Data analysis

Data of litter WR were submitted to Generalized Linear Mixed Model (GLMM) analysis, considering main and interactive effects of litter type (12 species), treated as a random effect, and litter age (0 and 180 days) treated as a fixed factor. Data from the microcosms experiment were analyzed by a further GLMM considering main and interactive effects of litter type and age as described above, plus incubation time as a fixed covariate, on SWR expressed as MED. Pairwise differences were tested using Tukey's HSD post-hoc test.

To address the relationships of LWR and SWR recorded at different incubation time with plant litter biochemistry three different approaches were considered. First, simple linear correlation analysis was separately tested between WR of litter and soil and each litter chemical parameter, including both elemental chemical parameters (i.e. N content, labile C, cellulose and lignin content, C/N ratio, Lignin/N ratio) and regions of the ^13^C-CPMAS NMR spectra selected from reference literature [24,31,37]. In a more detailed analysis, correlation was extensively tested between water repellency of the model sandy soil amended with the 24 litter types and ^13^C NMR data recorded for the same litter materials at each resonance signal (n = 190), providing a fine-resolution profile of the variation in C types in the tested litter material associated with the effect on SWR. This analysis allow to identify restricted ^13^C-CPMAS NMR spectral regions showing significant correlation with SWR [38]. The correlation was tested for statistical significance controlling for multiple comparisons, according to the false discovery rate (FDR) approach [38], at α = 0.01. Finally, a Principal Component Analysis (PCA) was carried out on a data matrix reporting, for each litter material, the values recorded for all chemical parameters. Vector data of water repellency recorded in soil samples amended with litter samples at different incubation times were also plotted in the PCA space as supplementary variables, following [39].

## Results

### Litter water repellency

**Fig 1:**
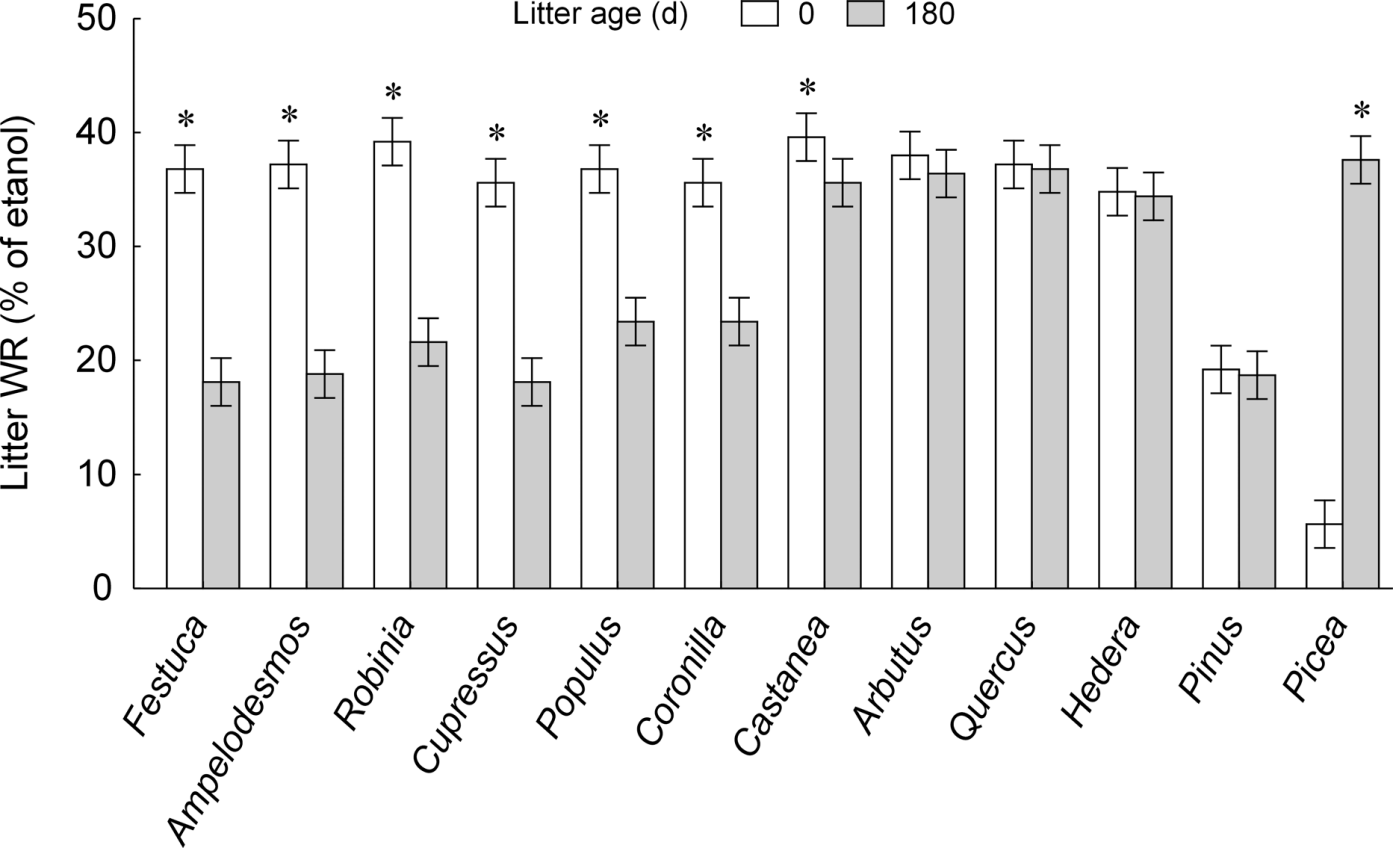
Observed water repellency in undecomposed and decomposed leaf litter. Litter water repellency in leaf litter types either undecomposed (0 days–open bars) and decomposed in litterbags for 180 days (filled bars). Data refer to mean and 95% confidence interval (N = 10 for each treatment combination) of MED, i.e. volume percentage concentration of ethanol in drops adsorbed by litter samples within 5 s. from administration. Plant litter species are ranked by decreasing difference between undecomposed and decomposed materials (significantly highest means within each plant litter species: *, *P* < 0.05, Tukey's HSD post-hoc test from the GLMM in S2 Table).

LWR largely varied according to litter type and age, which significantly interacted producing contrasting outcomes for different undecomposed and aged materials (Fig 1; S2 Table). However, in according to hydrophobicity classes proposed by Letey [35] and Schnabel et al. [20] (S1 Table), both undecomposed and aged litter were found to be highly water repellent (Fig 1). Considering undecomposed litter materials, LWR was extremely high for *Arbutus*, *Castanea* and *Robinia*, relatively low for *Picea* and *Pinus* and intermediate for the remaining species (Fig 1). Decomposition for 180 days differently affected LWR, with an increase of MED for *Picea*, not significant changes for *Arbutus*, *Quercus*, *Hedera* and *Pinus*, and a decrease, ranging from -50% to -10%, for the 7 remaining species (Fig 1). However, also in these latter cases aged litter were highly water repellent, with MED values of 18% or higher.

### Water repellency of soil amended with litter

**Fig 2:**
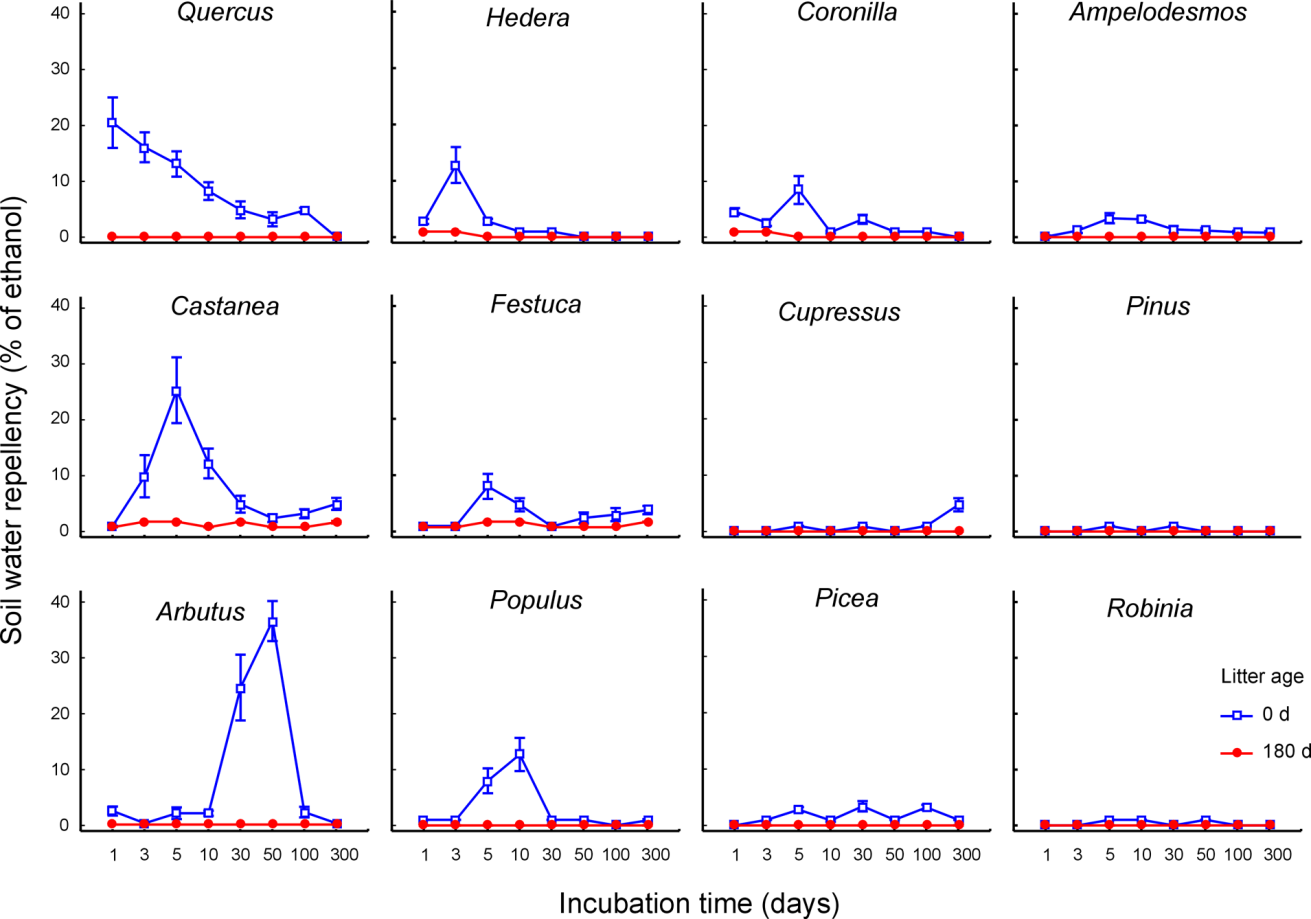
Water repellency of soil amended with litter at different incubation time. Dynamics of water repellency in a sandy loam soil amended with 24 different litter types (12 plant species at two ages, undecomposed and decomposed in litterbags for 180 days) over an incubation period of 300 days. Data refer to mean and 95% confidence interval (N = 10 replicates for each treatment combination) of MED, i.e. volume percentage concentration of ethanol in drops adsorbed by amended soil samples within 5 s. from administration.

In the microcosm experiment, all amending treatments (i.e. plant litter type, litter age and incubation time) largely affected SWR, with either main or interactive significant effects (S2 Table). The model sandy soil was not water repellent when unamended, showing MED values below 1% in all replicated controls. Differently, the application of plant litter in general enhanced SWR. However, occurrence and magnitude of the soil hydrophobic response were highly variable among the tested conditions, being significantly higher for amendments with fresh litter compared to aged materials (Fig 2 and significant main effect of litter age in S2 Table). Moreover, such pattern varied among the litter types, as indicated by the significant interactive effect of litter type and age (S2 Table). In particular, the addition of aged plant litter barely affected SWR, with a slight increase of MED recorded only for *Castanea*, *Coronilla*, *Festuca*, and *Hedera* (Fig 2), well below 3% in all cases. In contrast, the SWR increase after addition of fresh litter showed steep outbreaks for some specific materials (Fig 2), such as *Arbutus*, *Castanea* and *Quercus*, and lower levels when amended with *Cupressus*, *Picea*, *Pinus* and *Robinia* litter (Fig 2). Incubation time of amended soil samples greatly affected SWR, with both main and interactive effects in combination with litter age, and with both age and type (S2 Table). In other words, over the incubation period, soil treated with fresh litter showed variable response dynamics according to the litter species, with differences in times of SWR onset, peak and overall magnitude (Fig 2). In particular, fresh litter of *Coronilla*, *Hedera* and *Ampelodesmos* produced a rapid response, mostly peaking within 3–5 days from litter application. Also *Quercus* amendment show a similar behaviour, with a steep increase of SWR after litter application, followed by a progressive decrease (Fig 2). An early SWR onset was also observed for *Castanea* and *Festuca* amendments, but such treatments, as well as fresh *Cupressus* litter, also produced relative peaks of SWR at the end of the incubation period (Fig 2). In contrast, *Arbutus* and *Populus* litter produced a slower response, with maximum SWR after 50 and 10 days of incubation, respectively, followed by a rapid decrease (Fig 2). Finally, application of fresh litter from *Picea*, *Pinus* and *Robinia* had negligible effects on SWR (Fig 2).

### Relationships between soil water repellency and litter biochemistry

Considering plant litter materials, both elemental and proximate chemical parameters, as well as ^13^C-CPMAS NMR spectral regions, were not significantly associated with WR (Table 1). The highest correlation magnitude was recorded in the comparisons of LWR with ^13^C NMR N-alkyl and methoxyl C and H-C-substituted aromatic C regions, although in both cases the observed negative values were not statistically significant (Table 1).

**Table 1:** Linear correlation between LWR and litter biochemistry.

	Pearson coefficient and *P*-value (n = 24)	
**Elemental chemical parameters**		
Labile C (%)	+0.24	(*P* = 0.31)
Cellulose (%)	+0.09	(*P* = 0.71)
Lignin (%)	-0.21	(P = 0.38)
N content (%)	+0.09	(*P* = 0.70)
C: N ratio	+0.08	(*P* = 0.73)
Lignin: N ratio	-0.17	(*P* = 0.47)
^**13**^**C NMR-derived chemical parameters**		
Carboxylic C (161–190 ppm)	+0.24	(*P* = 0.30)
O-substituted aromatic C (141–160)	-0.10	(*P* = 0.66)
H-C-substituted aromatic C (111–140 ppm)	-0.39	(*P* = 0.08)
di-*O*-alkyl C (91–110 ppm)	+0.06	(*P* = 0.81)
*O*-alkyl C (61–90 ppm)	+0.13	(*P* = 0.59)
N-alkyl and methoxyl C (46–60 ppm)	-0.31	(*P* = 0.17)
Alkyl C (0–45 ppm)	-0.01	(*P* = 0.97)

Linear correlation (Pearson's *r* and associated *P-value*) between litter water repellency (LWR), as expressed by MED, and chemical parameters for 24 litter types (12 plant species, undecomposed and decomposed in litterbags for 180 days).

Concerning soil amended with litter materials, water repellency was differently associated to litter quality parameters, depending on incubation time (Table 2). Both labile C content and C/N ratio of litter showed a general trend of positive correlations with SWR, with significant values before 5 days and after 10 days of incubation, respectively. In contrast, lignin content and lignin/N ratio showed a negative association with SWR, which was significant after 3, 5 and 10 days of incubation in the first case and after 5 days for lignin/N ratio. Finally, no significant association was found between SWR and cellulose and N litter content.

**Table 2:** Linear correlation between SWR and litter biochemistry at different incubation time.

	Incubation time (days)							
	1	3	5	10	30	50	100	300
**Elemental chemical parameters**								
Labile C (%)	0.246	*0*.*419(0*.*042)*	*0*.*470(0*.*020)*	0.327	0.186	0.090	0.274	0.215
Cellulose (%)	0.153	0.186	0.153	0.292	0.172	0.168	0.358	0.307
Lignin (%)	-0.269	*-0*.*444(0*.*030)*	*-0*.*501(0*.*013)*	*-0*.*439(0*.*032)*	-0.201	-0.116	-0.376	-0.347
N content (%)	-0.16	-0.092	-0.003	-0.253	-0.238	-0.248	-0.328	-0.155
C: N ratio	0.292	0.302	0.343	*0*.*424(0*.*039)*	0.310	0.243	*0*.*577(0*.*003)*	0.352
Lignin: N ratio	-0.157	-0.393	*-0*.*451(0*.*027)*	-0.346	-0.049	0.018	-0.195	-0.287
^**13**^**C-CPMAS NMR regions**								
Carboxylic C (161–190 ppm)	-0.234	-0.216	-0.075	-0.141	-0.081	-0.020	-0.289	-0.033
O-substituted aromatic C (141–160 ppm)	0.008	-0.319	-0.073	0.070	0.223	0.287	0.343	0.302
H-C-substituted aromatic C (111–140 ppm)	0.009	-0.332	-0.115	0.040	0.209	0.264	0.297	0.124
di-*O*-alkyl C (91–110 ppm)	0.124	0.073	0.338	*0*.*419(0*.*041)*	0.185	0.164	*0*.*482(0*.*017)*	*0*.*491(0*.*015)*
*O*-alkyl C (61–90 ppm)	0.163	0.342	*0*.*462(0*.*023)*	*0*.*425(0*.*038)*	0.137	0.053	*0*.*399(0*.*054)*	*0*.*395(0*.*056)*
N-alkyl and methoxyl C (46–60 ppm)	-0.168	-0.252	*-0*.*439(0*.*032)*	*-0*.*478(0*.*018)*	-0.352	-0.312	*-0*.*574(0*.*003)*	*-0*.*564(0*.*004)*
Alkyl C (0–45 ppm)	-0.098	-0.100	-0.352	-0.375	-0.150	-0.113	*-0*.*407(0*.*049)*	-0.418*(0*.*042)*
*O*-alkyl C and di-*O*-alkyl C (61–110 ppm)	0,142	0,212	*0*,*448(0*.*028)*	*0*,*441(0*.*031)*	0,133	0,101	*0*,*499(0*.*013)*	*0*,*530(0*.*008)*
O- and H-C-substituted aromatic C (111–160 ppm)	0,000	-0,292	-0,155	0,008	0,231	0,269	0,220	0,018

Linear correlation between water repellency of a sandy soil (SWR) amended with 24 litter types (12 plant species, undecomposed and decomposed in litterbags for 180 days), assessed 8 times over an incubation period of 300 days, and chemical parameters of the litter materials. Data refer to Pearson's *r* and associated *P-value* (in brackets, only for significant correlation, marked in italics).

Considering biochemical litter quality from ^13^C-CPMAS NMR reference regions (Table 2), during the first 3 days of incubation SWR was not related to any class of C-types. However, at higher incubation time, SWR was positively associated with *O*-alkyl C and di-*O*-alkyl C regions, both at the intermediate (5 and 10 days) and later stages (100 and 300 days). In detail, the relationships between SWR and spectral data of the litter materials (S1 Fig) showed a significant correlation with signals resonating at 105–110 ppm and 75–90 ppm, respectively. In addition, the combination *O*-alkyl C and di-*O*-alkyl C fractions, which represent labile carbohydrate C, show a similar trend of correlation when related to SWR. Contrarily, an opposite correlation pattern was found for the N-alkyl and methoxyl C and alkyl C regions, with significant negative correlations after 5 and 10 days and at the latest stages (100 and 300 days) of incubation. In particular, negative correlation increased in magnitude with incubation time for most of alkyl C signals (0–30 ppm) and for all N-alkyl and methoxyl C signals (S1 Fig). Finally, the aromatic and carboxylic C regions were not associated with SWR.

**Fig 3:**
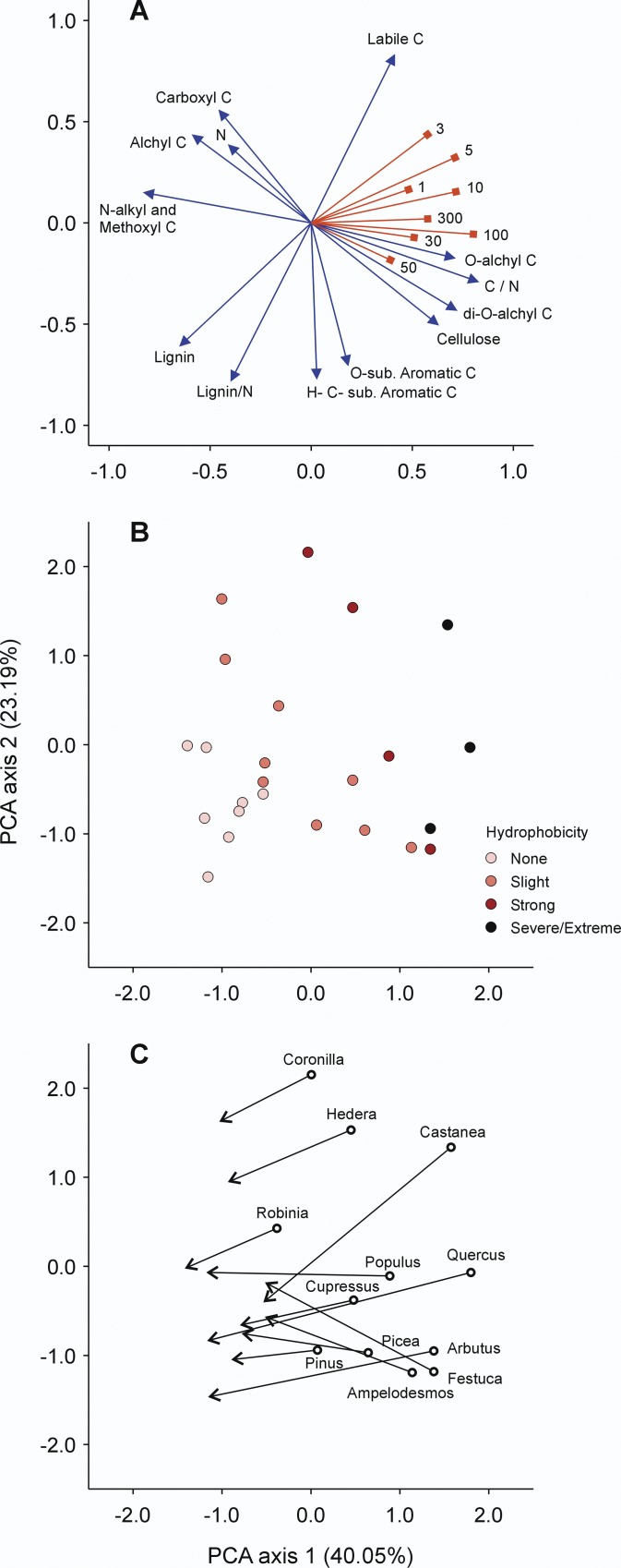
Principal component analysis ordination of biochemical parameters in litter samples and soil water repellency. Principal component analysis (PCA) ordination of biochemical parameters in 24 litter samples (12 plant species, undecomposed and after 180 days of decomposition) tested for production of WR in a sandy soil incubated for different time periods. (A) Loading vectors of litter parameters (^13^C NMR regions labelled by corresponding C types). Soil WR at different incubation periods (labelled by days of incubation) is also plotted as a supplementary variable following Legendre & Legendre [39]. (B) Factorial scores of litter samples represented according to maximum WR observed in soil during the incubation period, expressed in classes of hydrophobicity following Schnabel et al. [20]. (C) Decomposition trajectories of plant litter between 0 and 180 days, based on factorial scores of litter samples.

Principal component analysis (PCA) provided a satisfactory ordination of the biochemical quality parameters across litter types (Fig 3), both for ^13^C-NMR spectral regions derived from the literature and for elemental chemical parameters, with the first four eigenvalues accounting for 85.3% (40.0, 23.2, 12.8 and 9.4%) of the total variance. In Fig 3 are reported the loading vectors of litter quality parameters (i.e. for each ^13^C-NMR region, the relative abundance measured on each litter sample, while for elemental chemistry values actually recorded in litter materials, and how they relate to the PC axes), and the factorial scores of the 24 litter materials on the bi-dimensional space. The first two components show the individual litter sample spreading according to biochemical variations during the decomposition process and the associated effect on soil water repellency, and the related trajectories of the different litter species in the multivariate ordination space.

## Discussion

In this study we found that changes of litter biochemical quality during decomposition significantly affect SWR. Plant species and litter ages largely differ in the capability to induce SWR. Litter decomposed for 6 months, although hydrophobic *per se*, were barely capable to increase SWR when incorporated into the soil. On the contrary, undecomposed litter showed a strong capability to induce SWR, with a large variability observed among litter types, related to their biochemical quality. Surprisingly, we found that SWR induced by litter addition was unrelated to the aliphatic fraction of the litter substrates, while lignin poor but labile C rich substrates were the materials that most rapidly induced SWR, possibly related to their suitability as substrate for soil microbial activity. Finally, by defining litter quality with ^13^C-CPMAS NMR, our results provide a significant novel contribution towards a full understanding of the relationships between plant litter biochemistry and SWR.

### Litter water repellency

In previous works, plant litter has been considered as a source of hydrophobic substances capable to induce SWR with a different response depending on plant species [1,2,13]. Zavala et al. [17], in a comparative analysis on SWR under different Mediterranean vegetation types, reported a strong SWR under *Quercus suber*, *Pinus pinaster* and *Eucalyptus globulus*, suggesting the content of hydrophobic compounds in the plant leaf litter as a likely controlling factor. Moreover, in the same work, extremely high SWR was detected in soils under heathland plants, whereas most soils under olive trees resulted hydrophilic. Overall, it is quite surprising that most previous studies failed in presenting data on litter WR. Our data demonstrate that different plant litter types are consistently hydrophobic, with all herbaceous, evergreen and deciduous woody species showing a strong WR. In addition, we found that six months of microbial decomposition, in 58% of the cases (i.e. 7 out of 12 litter species) produced a significant decrease of litter WR that, however, remained quite hydrophobic. Since naturally-occurring hydrophobic compounds have typically organic origin [2,40], the observation that litter is highly water repellent irrespective of its decay stage is not surprising. Few previous studies investigated the relationships between litter biochemistry and WR [11]. In this perspective, considering the relatively high molecular diversity of plant litter, our approach based on ^13^C-CPMAS NMR allowed to highlight the biochemical differences among different undecomposed litter types and to assess the chemical changes occurring in such materials during the decomposition process [25,37]. In particular, considering reference ^13^C-CPMAS NMR spectral regions derived from the literature [24,33], the decomposition process produces significant changes consistent across different leaf materials, with a decrease of the relative fractions of *O*-alkyl C and an increase of alkyl C, N-alkyl C and methoxyl C, as previously reported for different study systems [26,29,34,41,42]. However, such consistent biochemical differences between fresh and decomposed litter, as well as differences in classic elemental chemical parameters, were hardly capable to explain the observed variability of litter WR among different litter materials. Indeed, the absence of correlation with litter WR, observed for all the tested regions and parameters, did not confirm the general pattern we could have expected. In detail, a supposed possible role of the degree of plant material lignification [43] was only marginally detected. In facts, considering ^13^C NMR regions including lignin spectral signals [24,31,34] a borderline correlation value was found between litter WR and the H-C-substituted aromatic C, while not significant values were observed for the N-alkyl and methoxyl C region (46–60 ppm) and proximate lignin content (Table 1). Even more surprisingly, litter WR was not associated with the alkyl C region of the ^13^C NMR spectra (0–45 ppm) which includes classes of aliphatic, hydrophobic C compounds most abundantly and frequently found in water repellent soils, such as alkanes and fatty acids [44–46]. However, in this regard, the partial reduction of WR observed for some litter types after the decomposition period should not be related to the whole alkylic region of plant litter spectra, because such fraction, as highlighted by a number of previous studies [26,41], significantly increases as microbial decomposition proceed, likely related to the build-up of lipidic metabolism and cell membranes of microbes feeding on decomposing litter. In more general terms, the limited ability of ^13^C NMR spectral regions as well as classic proximate chemical analyses to explain differences in litter WR could be due to two non-mutually exclusive reasons: i) the relatively low range of WR differences among litter types. Then, further studies comparing larger numbers of plant litter species will be potentially able to point out significant relationships between litter chemistry and WR. ii) the low resolution power of previous classifications of ^13^C NMR spectral regions, as related to the capability of discriminate between compounds differently affecting litter WR within each class of C types. Hence, further investigation of the relationships between litter WR and its biochemical quality as derived from ^13^C NMR data should be based on a finer spectral resolution, as in the case of our comparison with SWR.

### Relationships between litter chemistry and SWR

Our results demonstrate that when litter is incorporated into the soil, it can produce a variety of effects on SWR, ranging from a dramatic increase to a null effect depending on the considered litter type. Litter age appears the most relevant factor affecting SWR, with aged litter hardly capable to increase SWR in spite of the fact that such organic materials are highly water repellent *per se*. On the contrary, a large effect variability was produced by fresh litters, with soil response varying in SWR onset, peak and overall magnitude. Noteworthy, SWR was unrelated to litter WR (data not shown), with some of the most hydrophobic fresh litters (e.g. *Ampelodesmos*, *Cupressus*, *Robinia*) inducing only minor increases of SWR. This result, together with the observation that SWR was dynamically variable during the 300 days of microcosm incubation, suggest that SWR cannot be satisfactorily explained using information limited to litter biochemistry features but, rather, it is due to a combination of microbial and chemical-physical processes occurring into the soil. Our results indicate that immediately after the soil amendment, i.e. before microbial decomposition starts, the direct abiotic effect of litter hydrophobic compounds is limited, so that chemical-physical interactions between litter hydrophobic compounds and soil particles, in order to produce SWR, are likely mediated by biological processes. The fresh litter of *Quercus*, inducing a steep SWR increase the day after its application, was the only exception to such response. In this case, the molecules contained in *Quercus* litter may have directly interacted with soil particles, producing SWR by a direct physical-chemical action. The underlying mechanisms could be explained by adsorption and inter-particle coating by hydrophobic domains of litter compounds. However, only few authors have monitored the changes of soil aggregate stability following the addition of organic matter and other materials, such as ash (but see [47]).

In contrast with evidence of direct abiotic effects, several hydrophobic compounds of biological origin have been recognised as potentially responsible for SWR. As an example, a comparative analysis of some potential biological sources of soil hydrophobic properties and other chemical features of Australian sands [11] demonstrated that polar waxes extracted from *Eucalyptus* leaf litter and bark, as well as other plant materials, were chemically and hydrophobically closer to waxes from sands with respect to waxes isolated from fungi and actinomycetes. Such similarities, in relation to WR, mostly relied on unbranched and branched C16 to C36 fatty acids and their esters, alkanes, phytanols, phytanes, and sterols, some of which were not detected in the microbial waxes. In many studies, searching for the molecular determinants of SWR, different organic fractions were characterized after extraction from water repellent soils (e.g. [40]) or wettable soils were treated with molecules identified in such extracts (e.g. [48]), highlighting the contributions of polar compounds of high molecular mass as necessary for SWR, and suggesting that it depends more on the presence of specific compounds rather than their quantity.

Our results indicate that, for 11 out of 12 undecomposed plant litter types, the peak in SWR, or the absence of SWR compared to plain litter, are likely related to microbial decomposing agents either producing unspecified hydrophobic compounds in the soil-litter mixture or disrupting hydrophobic domains formerly occurring in litter materials. Both phenomena have been previously reported and attributed to different biological processes. The disruption of hydrophobic compounds by soil microorganisms has been observed in the case of wax-degrading bacteria and actinomycetes [49] and was proposed for exploitation in bioremediation applications [50]. Analogous processes could explain our observations for soil treated with the litters of *Robinia*, *Pinus*, *Picea* and *Cupressus*, that mostly showed negligible SWR occurrence during the incubation period. Moreover, the relative water repellency of such litter materials were highly variable, ranging from the extreme hydrophobicity of fresh leaves of *Robinia* and *Cupressus* and decomposed litter of *Picea* to the strong hydrophobicity of *Pinus* litter and the slight hydrophobicity of fresh *Picea* needles, indicating that for such materials the neutralization of initial hydrophobicity after incubation in soil was unrelated to the initial condition.

On the other hand, the emergence of soil hydrophobicity in soil by bacterial activity was previously investigated (review in [51]), in relation to the biosynthesis of extracellular polymeric substances (EPS) implicated in the formation of bacterial biofilms [52], which can become hydrophobic when dry, and processes of soil bioclogging from microorganism overgrowth. Such previous evidence is consistent with our observations for soil treated with lignin poor, fast decomposing litter (i.e. *Castanea*, *Coronilla*, *Hedera*, *Populus* and, to a lesser extent, for *Ampelodemsmos*), all showing SWR onset within 3–10 days after the litter application followed by a rapid decrease. Indeed such SWR dynamics are compatible with a consistent microbial growth after feeding on labile C compounds, up to a threshold limit corresponding to biogenic hydrophobicity by overclogging. Similarly, the delayed SWR peaks observed for soil treated with litter of *Arbutus*, could be related to a retarded microbial growth plateau owing to the lower content of labile C and the higher content of lignin of such plant material [29].

The intrinsic initial biochemical characteristics of plant litter are major factors driving its decomposition rate [26,53,54], hence directly or indirectly affecting interconnecting ecological processes, including litter nutrient dynamics [55–57] and suitability for microbial feeding [58–60], phytotoxicity [29,61] and plant-soil negative feedback [27]. Surprisingly, few studies attempted to relate the biochemical characteristics of different organic products with their effect on SWR. Here, we showed that the initial biochemical characteristics of organic matter are suitable to explain the variability of SWR after litter input, as related to litter capability of sustaining microbial growth. In particular, SWR is positively associated with the abundance of labile, easily decomposable C fractions mainly composed of carbohydrates (NMR spectral regions corresponding to di-*O*-alkyl C and *O*-alkyl C), as well as with the labile C fraction determined by proximate chemical analysis. These results provide further support to our hypothesis of a rapid burst of microbial activity, sustained by the high availability of sugars and labile C compounds for soil treated with some undecomposed litter types (i.e. *Castanea*, *Coronilla*, *Hedera*, and *Populus*) leading to rapid SWR onset. Besides, for such materials, the rapid SWR decrease after peaking suggests not only that microbes may produce strongly water repellent compounds, but also that these compounds might be short–lived and rapidly subjected to chemical or microbial breakdown. Interestingly, we found a significant positive association between SWR and restricted spectral regions resonating at 105–110 ppm and at 75–90 ppm, which emerged and progressively increased over incubation time. Such results are unclear, but possibly related to the late onset of slight SWR in soils treated with undecomposed litters of *Castanea*, *Festuca*, *Cupressus* and, toa lesser extent, of *Populus* and *Picea*. A possible, although speculative hypothesis, is that the residual availability at long term of specific carbohydrates, might sustaining microbial succession over these materials.

A different behaviour was found for lignin-rich, labile C-poor materials. In this respect, previous studies based on litter proximate chemical analyses coupled with ^13^C-CPMAS NMR data highlighted that, as decomposition proceeds, a sharp decrease of the labile C fraction and a relative increase of lignin and lignin-like aromatic compounds are expected [26]. In detail, previous ^13^C-CPMAS NMR analysis [26] of some litter tested here showed a rapid reduction of carbohydrates (spectral regions corresponding to di-*O*-alkyl C and *O*-alkyl C) during litter decomposition. Here, we found consistent negative correlations between SWR and NMR regions related to lignin and proteins and peptides (methoxyl and N-alkyl C region, 46–60 ppm) as well as with lignin and lignin/N ratio. These results can be related to the effect of the lignin rich and labile C-poor aged litter on SWR. Decomposed litter is unsuitable to sustain microbial growth, and can even inhibit microbes by the presence of recalcitrant and/or fungitoxic compounds [60]. Hence, our hypothesis is that aged litter is unable to induce SWR because do not support a substantial microbial growth. Finally, it is surprising that the aliphatic fraction of the NMR spectra (Alkyl C, 0–45 ppm) was not associated with SWR, with a restricted region (0–30 ppm) even negatively correlated to soil hydrophobicity at long term. These results, in contrast with some previous finding [60], suggest that hydrophobic compounds initially present in plant tissues play a negligible role in SWR, while the result at long term might be related to the abundance of waxes in conifer litter, showing high spectral intensity in the 0–30 ppm region and not inducing SWR. Further investigation is needed to explicitly test this hypothesis.

## Conclusions

Our multi-species bioassay approach revealed that plain plant litters with similar WR has species-specific effect on SWR. Noteworthy, we found that undecomposed plant litter often induces a rapid increase of SWR likely acting as a C source for saprophytic microbes. An opposite response was found for soil treated with aged litter that, even when highly hydrophobic *per se*, barely affects SWR when incorporated into the soil. The use of ^13^C-CPMAS NMR provides an improved definition of litter biochemical quality, helping to explain the variable effects of plant litter on SWR. In detail, ^13^C-CPMAS NMR revealed that the two restricted *O*-alkyl C and N-alkyl and methoxyl C spectral regions are crucial to understand litter effects, with SWR enhanced by labile C-rich materials but negatively associated with signals related to plant tissue lignifications. We are aware that the implications of our findings for the understanding of SWR dynamics in natural soil-plant systems are limited by the use of only 24 litter types and a single soil type. However, as a major novel contribution, our study is the first attempt to linking litter biochemistry with dynamics of SWR.

## Supporting Information

S1 Fig Correlation profiles between soil water repellency and ^13^C-CPMAS NMR spectral signals of litter at different incubation time. Correlation profiles (Pearson’s *r*) between water repellency of a sandy soil amended with 24 litter types (12 plant species, undecomposed and decomposed in litterbags for 180 days) and incubated for 300 days and ^13^C-CPMAS NMR spectral signals of the litter samples (n = 24). Red dashed lines indicate threshold values of statistical significance for *r* (P < 0.01 after correction for multiple comparisons according to the false discovery rate method [38]).
(TIF)

S1 Table Correspondence between % ethanol and hydrophobicity classes after Letey [35] and Schnabel et al. [20]. (DOCX)

S2 Table Summary of the GLMM of the water repellency experiments. Summary of the generalized linear mixed models (GLMM) testing for main and interactive effects of treatments on MED (i.e. volume percentage concentration of ethanol in drops adsorbed by litter samples within 5 s. from administration) in water repellency experiments. The model for litter WR includes first and second order effects of litter type (L, treated as a random factor) and litter age (A, treated as a fixed factor with two levels, either undecomposed or decomposed for 180 days). The model for soil WR, in addition to such effects, includes litter incubation time in soil (T, treated as a fixed covariate) and interactions of L, A and T.
(DOCX)
