# Supplementary figures and images for: The Influence of Plant Litter on Soil Water Repellency: Insight from 13C NMR Spectroscopy
Source: PLoS One. 2016 Mar 29;11(3):e0152565. doi: 10.1371/journal.pone.0152565 (PMC4811566; doi:10.1371/journal.pone.0152565)

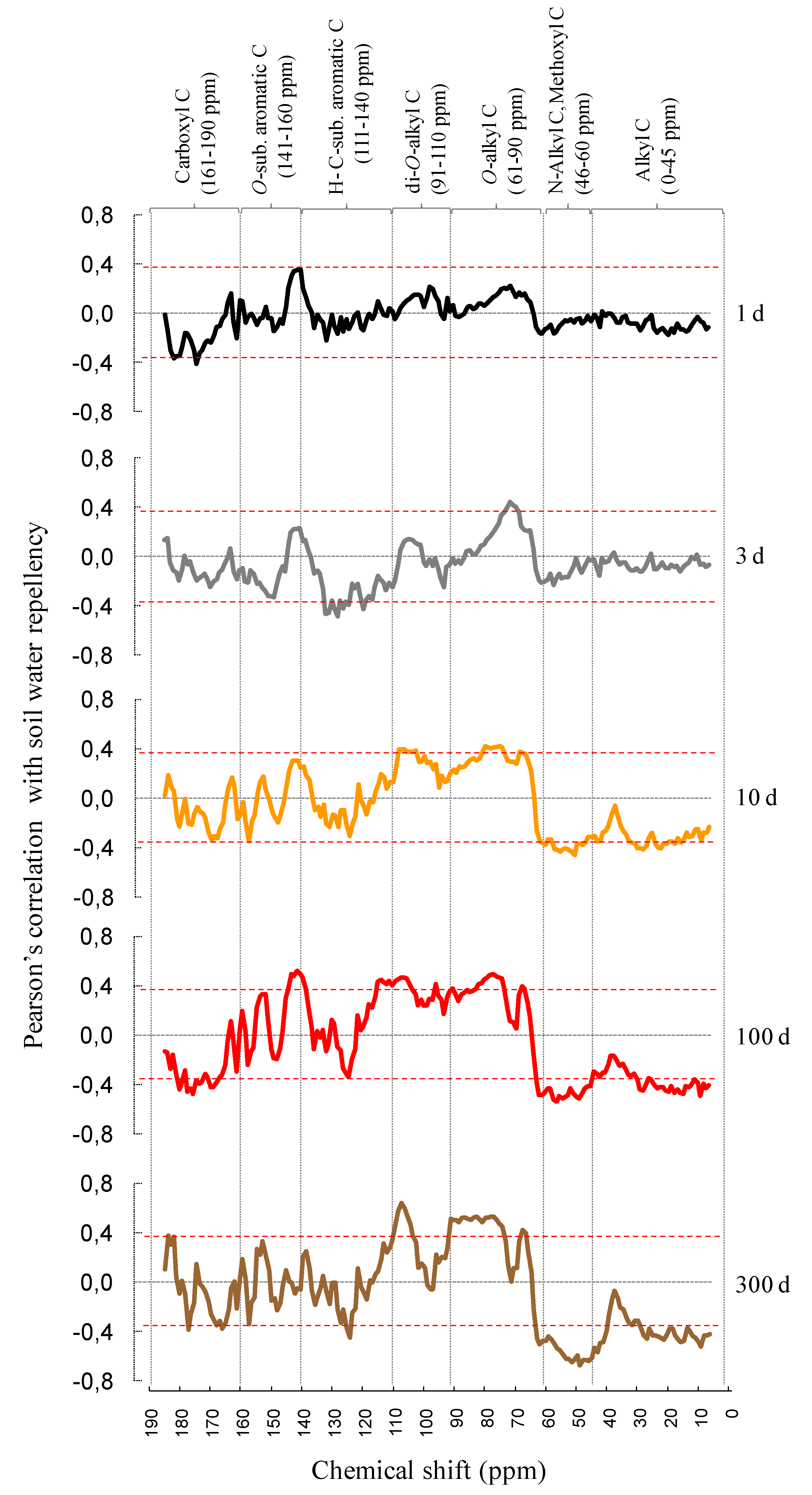

Supplement: S1 Fig — Correlation profiles (Pearson’s r) between water repellency of a sandy soil amended with 24 litter types (12 plant species, undecomposed and decomposed in litterbags for 180 days) and incubated for 300 days and 13C-CPMAS NMR spectral signals of the litter samples (n = 24). Red dashed lines indicate threshold values of statistical significance for r (P < 0.01 after correction for multiple comparisons according to the false discovery rate method [38]). (TIF) [file pone.0152565.s001.TIF]
