# Supplementary material for: The Influence of Plant Litter on Soil Water Repellency: Insight from 13C NMR Spectroscopy
Source: PLoS One. 2016 Mar 29;11(3):e0152565. doi: 10.1371/journal.pone.0152565 (PMC4811566; doi:10.1371/journal.pone.0152565)
Supplement: S1 Table — (DOCX) [file pone.0152565.s002.docx]

**S1 Table. Correspondence between % ethanol and hydrophobicity classes after Letey [35] and Schnabel et al. [20].**

| **Ethanol %** | **Hydrophobicity class** | **Severity** |
| --- | --- | --- |
| 0 | 1 | Hydrophilic |
| 1 | 2 | Slightly hydrophobic |
| 3 | 3 | Slightly hydrophobic |
| 5 | 4 | Slightly hydrophobic |
| 8,5 | 5 | Strongly hydrophobic |
| 13 | 6 | Strongly hydrophobic |
| 18 | 7 | Severely hydrophobic |
| 24 | 8 | Severely hydrophobic |
| 36 | 9 | Extremely hydrophobic |
| > 36 | 10 | Extremely hydrophobic |
