# Supplementary material for: The Influence of Plant Litter on Soil Water Repellency: Insight from 13C NMR Spectroscopy
Source: PLoS One. 2016 Mar 29;11(3):e0152565. doi: 10.1371/journal.pone.0152565 (PMC4811566; doi:10.1371/journal.pone.0152565)
Supplement: S2 Table — Summary of the generalized linear mixed models (GLMM) testing for main and interactive effects of treatments on MED (i.e. volume percentage concentration of ethanol in drops adsorbed by litter samples within 5 s. from administration) in water repellency experiments. The model for litter WR includes first and second order effects of litter type (L, treated as a random factor) and litter age (A, treated as a fixed factor with two levels, either undecomposed or decomposed for 180 days). The model for soil WR, in addition to such effects, includes litter incubation time in soil (T, treated as a fixed covariate) and interactions of L, A and T. (DOCX) [file pone.0152565.s003.docx]

**S2 Table. Summary of the GLMM of the water repellency experiments.**

|  | **Effect type** | **SS** | **df** | **MS** | **F** | **p** |
| --- | --- | --- | --- | --- | --- | --- |
| **Litter WR** |  |  |  |  |  |  |
| Litter_type (L) | Random | 7856.1 | 11 | 714.2 | 0.704 | 0.7148 |
| Litter age (A) | Fixed | 2205.2 | 1 | 2205.2 | 2.174 | 0.1684 |
| L × A | Random | 11157.7 | 11 | 1014.3 | 89.837 | < 0.0001 |
| **Soil WR** |  |  |  |  |  |  |
| Litter_type (L) | Random | 5384.1 | 11 | 489.47 | 1.031 | 0.4780 |
| Litter age (A) | Fixed | 5642.3 | 1 | 5642.31 | 11.890 | 0.0054 |
| Incubation time (T) | Fixed | 486.9 | 1 | 486.97 | 5.230 | 0.0430 |
| L × A | Random | 5220.1 | 11 | 474.55 | 34.251 | < 0.0001 |
| L × T | Random | 1024.1 | 11 | 93.10 | 1.040 | 0.4747 |
| A × T | Fixed | 476.7 | 1 | 476.67 | 5.324 | 0.0415 |
| L × A × T | Random | 984.9 | 11 | 89.54 | 6.462 | < 0.0001 |

Summary of the generalized linear mixed models (GLMM) testing for main and interactive effects of treatments on MED (i.e. volume percentage concentration of ethanol in drops adsorbed by litter samples within 5 s. from administration) in water repellency experiments. The model for litter WR includes first and second order effects of litter type (L, treated as a random factor) and litter age (A, treated as a fixed factor with two levels, either undecomposed or decomposed for 180 days). The model for soil WR, in addition to such effects, includes litter incubation time in soil (T, treated as a fixed covariate) and interactions of L, A and T.
